# Association between *TNF, IL1B, IL6, IL10* and *IFNG* polymorphisms and recurrent miscarriage: a case control study

**DOI:** 10.1186/s12958-017-0300-3

**Published:** 2017-10-10

**Authors:** Jianting Ma, Xingguang Zhang, Gang He, Chunlin Yang

**Affiliations:** Department of Obstetrics and Gynecology, the People’s Hospital of Yuyao City, East Road No.800, Yuyao, Zhejiang Province 315400 China

**Keywords:** Cytokine, Polymorphism, Pregnancy loss, Recurrent miscarriage

## Abstract

**Background:**

Approximately half of recurrent miscarriages have unexplained etiology. Recent evidences suggest that cytokines are important determinants in pregnancy maintenance and as such, cytokine gene polymorphisms, which can affect cytokine production and/or functionality, could play a role in the disorder. Thus, we aimed to investigate the association of selected cytokine gene polymorphisms with risk of recurrent miscarriage among Chinese.

**Methods:**

*TNF* -238G > A, *TNF* -308G > A, *IL1B* -511 T > C, *IL1B* 3954C > T, *IL6* -174G > C, *IL6* -634C > G, *IL10* -1082A > G and *IFNG* 874A > T polymorphisms were genotyped on 775 women with idiopathic recurrent miscarriage and 805 healthy parous control women. Logistic regression analysis was performed to determine the odds ratios (ORs) of the association between the polymorphisms and recurrent miscarriage risk.

**Results:**

Among the eight polymorphisms studied, only the *IL1B* -511 T > C and *IL6* -634C > G polymorphisms showed statistically significant associations with recurrent miscarriage risk. For the former, a significantly increased risk of recurrent miscarriage was observed for the mutant (CC) genotype (OR: 1.377; 95% CI: 1.039–1.824; *P* = 0.026). However, for the *IL6* -634C > G polymorphism, a decreased recurrent miscarriage risk was observed for the heterozygous (CG) genotype (OR: 0.614; 95% CI: 0.493–0.765; *P* < 0.001) and the mutant (GG) genotype (OR: 0.414; 95% CI: 0.251–0.684; *P* = 0.001).

**Conclusions:**

The *IL1B* -511 T > C polymorphism may serve as important risk factor for recurrent miscarriage while the *IL6* -634C > G polymorphism may protect against the risk of recurrent miscarriage.

## Background

Recurrent miscarriage refers to the consecutive loss of pregnancy before the 20th gestational week for three or more times [1]. This event affects approximately 1–2% of otherwise healthy females, and poses a significant physical and emotional burden on women experiencing the incidence and their family members [2]. A number of etiological factors have been described for recurrent miscarriages, including genetic defects, anatomical abnormalities, endocrinologic disorders, infections, thrombophilias, and environmental exposures [2]. However, approximately 50% of recurrent miscarriages are not attributable to these etiological factors and their causes remained unexplained [1].

Recently, the contributions of immunogenetic factors to the etiopathology of recurrent miscarriages have been suggested [3]. During early stages of normal pregnancies, pro-inflammatory T helper (Th) 1 cytokines are necessary for stimulating vasculogenesis which is essential for a successful embryonic implantation [4]. However, a prolonged exposure to Th1 cytokines can result in a cell-mediated immune response that is detrimental to the fetus, resulting in pregnancy losses [5]. As such, during later stages of successful pregnancies, a shift from pro-inflammatory Th1 immunity to anti-inflammatory Th2 immunity is commonly observed. The proper homeostatic balance between Th1 and Th2 cytokines is critically important for a stable maintenance of pregnancy and other reproductive events [6, 7]. It has been observed that normal pregnancies are accompanied by an overall higher expression of Th2 cytokines while pregnancies with unfavorable outcomes are usually associated with an overall higher expression of Th1 cytokines [8].

The expression of cytokines, as well as the immune response elicited, could be influenced by polymorphisms in cytokine genes. Thus, cytokine gene polymorphisms could potentially influence the risk of recurrent miscarriage. In this study, we investigated the association of eight selected cytokine gene polymorphisms with risk of recurrent miscarriage among Chinese women. The cytokine gene polymorphisms selected were *TNF* -238G > A, *TNF* -308G > A, *IL1B* -511 T > C, *IL1B* 3954C > T, *IL6* -174G > C, *IL6* -634C > G, *IL10* -1082A > G and *IFNG* 874A > T. All the cytokines studied are Th1 cytokines, except IL10, which shows dual (inhibitory and stimulatory) immunologic functions and thus could not be classified as either Th1 or Th2 [9]. All the polymorphisms included in this work have been previously investigated in relation to their association with the risk of recurrent miscarriage in a number of populations, and significant findings have been observed in a few instances [10–19]. However, population-to-population differences are common in genetic association studies, and the association of these cytokine gene polymorphisms with the risk of recurrent miscarriage has not been extensively studied among Chinese women, especially those from the Eastern China population. This study aimed to address this insufficiency of data in the Eastern China population.

## Methods

### Ethics, consent and permissions

The study was approved by the Institutional Review Board of People’s Hospital of Yuyao City, Zhejiang Province (approval number: 20,110,030,024). The study was conducted in accordance to the Declaration of Helsinki. Written informed consent was obtained from all participants before being included in the study.

### Participants

All young women (age ≤ 40 years) with recurrent miscarriages were identified from the obstetrical department of the People’s Hospital of Yuyao City, Zhejiang Province between December 2011 and January 2017. All women were at active (non-menopause) phase of menstruation. Recurrent miscarriages were defined as having a minimum of three clinically detectable spontaneous abortions before the 20th gestational week. Women with a history of diabetes or hypertension, drug or alcohol abuse, and smoking were excluded. Of the 2038 eligible women, 1742 agreed to participate in the study. A number of tests were performed to identify the causes of recurrent miscarriage among all these women, including hysterosalpingography, ultrasound, parental karyotypic analysis, examination of luteal phase defects, measurements of anti-phospholipid antibodies and lupus anticoagulants, examination of TORCH infections, tests of autoimmune disorders and erythroblastosis fetalis (Rh incompatibility) and other in-house examinations. In some but not all women, additional tests such as saline-infusion sonohysterography, hysteroscopy or endocrine tests were also performed. After exclusion of the participants with known causes of recurrent miscarriages, 775 patients were classified as being idiopathic and were recruited into the study. Meanwhile, 805 healthy parous women were recruited as controls. Controls were women who had at least 3 successful live births, with no history of miscarriages or preterm birth, and aged-matched to the cases. At recruitment, the interval from the last event (miscarriage or delivery) of the women ranged from 3 to 11 months (mean: 7.36 months). All participants were Han Chinese in ethnicity. A total of 5 ml blood specimen was collected from each participant for genetic analysis. Only 200 μl of the blood was eventually used, and the remaining was stored in our institution bio-repository for future use.

### Genotyping

Genotyping was performed on genomic DNA derived from the 200 μl blood specimens of the participants. Genomic DNA was isolated with the ethanol precipitation method using TIANamp Blood DNA kit (Beijing, China). Briefly, blood sample from each participant was sequentially mixed with 20 μl Proteinase K and 200 μl Buffer GB, and incubated at 56 °C for 10 min. After a homogenous mixture was formed, 200 μl absolute ethanol was added to precipitate the DNA. The mixture was transferred to a Spin Column CB3 and centrifuged at full speed for 30 s. The spin column was then washed sequentially with 500 μl Buffer GD and 600 μl Buffer PW and centrifuged at full speed for 30 s and 2 min respectively. After the washing step, the DNA was eluted at 100 μl Buffer TB and used for genotyping of the polymorphisms.

All polymorphisms were genotyped by polymerase chain reaction restriction fragment length polymorphism (PCR-RFLP) assays, except the *IFNG* 874A > T polymorphism, which was genotyped by allele-specific PCR. All PCR reactions were performed with Tiangen Fast HiFidelity PCR Kit (Beijing, China) on a Bioer Life Express thermocycler (Zhejiang, China). The PCR primers used for genotyping of the polymorphisms are shown in Table 1. Similarly, the restriction enzyme used and the band sizes obtained are also shown in Table 1. Roughly 10% of the PCR products were chosen at random and sequenced by using Applied Biosystems BigDye Terminator v3.1 Cycle Sequencing Kit (Foster City, California, United States) to independently confirm the genotypes.

**Table 1:** Primers used for genotyping

Polymorphism	Primers	Annealing temp (°C)	Restriction enzyme	Band sizes (bp)
*TNF* -238G > A	F: 5′-AAA CAG ACC ACA GAC CTG GTC-3′	59	BamHI	G: 130, 20
	R: 5′-CTC ACA CTC CCC ATC CTC CCG GAT C-3′			A: 150
*TNF* -308G > A	F: 5′-GAG GCA ATA GGT TTT GAG GGC CAT-3′	58	NcoI	G: 87, 20
	R: 5′-GGG ACA CAC AAG CAT CAA G-3’			A: 107
*IL1B* -511 T > C	F: 5′-TGG CAT TGA TCT GGT TCA TC-3’	58	AvaI	T: 305
	R: 5′-GTT TAG GAA TCT TCC CAC TT-3’			C: 190, 115
*IL1B* 3954C > T	F: 5′-GTT GTC ATC AGA CTT TGA CC-3’	54	TaqI	T: 250
	R: 5′-TTC AGT TCA TAT GGA CCA GA-3’			C: 136, 114
*IL6* -174G > C	F: 5′-GGA GTC ACA CAC TCC ACC T-3’	59	SfaNI	G: 532
	R: 5′-GTG GGG CTG ATT GGA AAC C-3’			C: 474, 58
*IL6* -634C > G	F: 5′-GAG AGG CCT TGA AGT AAC TG-3’	58	BsrBI	C: 180
	R: 5′-AAC CAA AGA TGT TCT GAA CTG A-3’			G: 120, 60
*IL10* -1082A > G	F: 5′-GTA AGC TTC TGT GGC TGG AGT-3’	60	MnlI	A: 130
	R: 5′-TTT CCA GAT ATC TGA AGA AGT CCT G-3’			G: 100, 30
*IFNG* 874A > T	F: 5′-TCA ACA AAG CTG ATA CTC CA-3’	61	–	261
	R1: 5′-TTC TTA CAA CAC AAA ATC AAA TCA-3’			
	R2: 5′-TTC TTA CAA CAC AAA ATC AAA TCT-3’			

### Statistics

Frequencies of the polymorphic genotypes were calculated by direct counting. Differences in the genotype frequencies between cases and controls were evaluated with chi-squared analysis. Each polymorphism was evaluated for possible deviation from the Hardy-Weinberg equilibrium by using a goodness-of-fit test. Logistic regression analysis was done to calculate the odds ratios (ORs) of the risk of recurrent miscarriage contributed by the polymorphisms. All statistical tests were two-sided and were performed with SPSS software version 19. All comparisons were considered to be statistically significant at *P* < 0.05.

## Results

The eight polymorphisms were successfully genotyped in all 775 cases and 805 controls. All results obtained from initial genotyping methods (PCR-RFLP or allele specific PCR) agreed with the independent confirmatory sequencing reactions. The frequencies of the polymorphic genotypes among the study participants are shown in Table 2. Statistically significant difference in the distribution of genotypes between the cases and controls was only observed for the *IL6* -634C > G polymorphism (*P* < 0.001), with the wild type (CC) genotype observed to be overrepresented in the cases and the heterozygous (CG) and mutant (GG) genotypes observed to be overrepresented in the controls. The CC, CG and GG genotypes were seen in 554 (71.5%), 197 (25.4%) and 24 (3.1%) of the cases, and 478 (59.4%), 277 (34.4%) and 50 (6.2%) of the controls, respectively.

**Table 2:** Distribution of genotypes between cases and controls

Polymorphism	Case (*N* = 775)	Control (*N* = 805)	*P*
*TNF* -238G > A			0.308
GG	732 (94.5%)	745 (92.5%)	
GA	41 (5.3%)	57 (7.1%)	
AA	2 (0.3%)	3 (0.4%)	
*TNF* -308G > A			0.307
GG	683 (88.1%)	726 (90.2%)	
GA	86 (11.1%)	76 (9.4%)	
AA	6 (0.8%)	3 (0.4%)	
*IL1B* -511 T > C			0.079
TT	213 (27.5%)	257 (31.9%)	
TC	384 (49.5%)	392 (48.7%)	
CC	178 (23.0%)	156 (19.4%)	
*IL1B* 3954C > T			0.777
CC	602 (77.7%)	632 (78.5%)	
CT	168 (21.7%)	166 (20.6%)	
TT	5 (0.6%)	7 (0.9%)	
*IL6* -174G > C			0.135
GG	484 (62.5%)	463 (57.5%)	
GC	248 (32.0%)	291 (36.1%)	
CC	43 (5.5%)	51 (6.3%)	
*IL6* -634C > G			**< 0.001**
CC	554 (71.5%)	478 (59.4%)	
CG	197 (25.4%)	277 (34.4%)	
GG	24 (3.1%)	50 (6.2%)	
*IL10* -1082A > G			0.186
AA	683 (88.1%)	685 (85.1%)	
AG	88 (11.4%)	113 (14.0%)	
GG	4 (0.5%)	7 (0.9%)	
*IFNG* 874A > T			0.589
AA	613 (79.1%)	621 (77.1%)	
AT	154 (19.9%)	173 (21.5%)	
TT	8 (1.0%)	11 (1.4%)	

It is also noteworthy to mention that although the genotype distribution of the *IL1B* -511 T > C polymorphism did not differ significantly between the cases and controls, the *P* value was at the borderline of statistical significance (*P* = 0.079). Contrary to the *IL6* -634C > G polymorphism, the wild type (TT) genotype of the *IL1B* -511 T > C polymorphism was found at a higher frequency in the controls, while the heterozygous (TC) and mutant (CC) genotypes were observed more frequently in the cases. The TT, TC and CC genotypes were respectively present in 213 (27.5%), 384 (49.5%) and 178 (23.0%) of the cases, and 257 (31.9%), 392 (48.7%) and 156 (19.4%) of the controls.

A clear lack of statistical significance was observed for the genotype distribution of the remaining polymorphisms, with *P* values of 0.308 (*TNF* -238G > A polymorphism), 0.307 (*TNF* -308G > A polymorphism), 0.777 (*IL1B* 3954C > T polymorphism), 0.135 (*IL6* -174G > C polymorphism), 0.186 (*IL10* -1082A > G polymorphism) and 0.589 (*IFNG* 874A > T polymorphism), respectively.

The genotype distribution of all the eight polymorphisms conformed to the Hardy-Weinberg equilibrium in both cases and controls (Table 3).

**Table 3:** Lack of deviation of the genotype distribution from HWE

Polymorphism	*P* value of HWE deviation in cases	*P* value of HWE deviation in controls
*TNF* -238G > A	0.066	0.098
*TNF* -308G > A	0.078	0.506
*IL1B* -511 T > C	0.845	0.766
*IL1B* 3954C > T	0.065	0.277
*IL6* -174G > C	0.136	0.562
*IL6* -634C > G	0.211	0.249
*IL10* -1082A > G	0.611	0.334
*IFNG* 874A > T	0.627	0.789

Significant data is captured in bold

Next, logistic regression analysis was performed to find out the odds ratio (OR) of each polymorphic genotype in contributing to the risk of recurrent miscarriage. The results were presented in Table 4. It was shown that statistically significant ORs were only observed the mutant (CC) genotype of the *IL1B* -511 T > C polymorphism (*P* = 0.026), as well as the heterozygous (CG) and mutant (GG) genotypes of the *IL6* -634C > G polymorphism (*P* < 0.001 and *P* = 0.001 respectively). The mutant (CC) genotype of the *IL1B* -511 T > C polymorphism was associated with an increased risk of recurrent miscarriage, with an OR of 1.377 (95% confidence interval (CI): 1.039–1.824). On the contrary, a decreased risk of recurrent miscarriage was noted for the heterozygous (CG) and mutant (GG) genotypes of the *IL6* -634C > G polymorphism, which showed an OR of 0.614 (95% CI: 0.493–0.765) and 0.414 (95% CI: 0.251–0.684) respectively.

**Table 4:** Odds ratio of the association between the polymorphisms and risk of recurrent miscarriage

Polymorphism	Odds ratio (OR)	*P*
*TNF* -238G > A		
GG	Reference	
GA	0.732 (95% CI: 0.484–1.108)	0.140
AA	0.679 (95% CI: 0.113–4.073)	0.671
*TNF* -308G > A		
GG	Reference	
GA	1.203 (95% CI: 0.868–1.666)	0.267
AA	2.126 (95% CI: 0.530–8.534)	0.288
*IL1B* -511 T > C		
TT	Reference	
TC	1.182 (95% CI: 0.939–1.487)	0.154
CC	1.377 (95% CI: 1.039–1.824)	**0.026**
*IL1B* 3954C > T		
CC	Reference	
CT	1.063 (95% CI: 0.834–1.353)	0.623
TT	0.750 (95% CI: 0.237–2.376)	0.625
*IL6* -174G > C		
GG	Reference	
GC	0.815 (95% CI: 0.660–1.008)	0.059
CC	0.807 (95% CI: 0.527–1.234)	0.322
*IL6* -634C > G		
CC	Reference	
CG	0.614 (95% CI: 0.493–0.765)	**< 0.001**
GG	0.414 (95% CI: 0.251–0.684)	**0.001**
*IL10* -1082A > G		
AA	Reference	
AG	0.781 (95% CI: 0.580–1.052)	0.104
GG	0.573 (95% CI: 0.167–1.967)	0.376
*IFNG* 874A > T		
AA	Reference	
AT	0.902 (95% CI: 0.706–1.151)	0.407
TT	0.737 (95% CI: 0.294–1.844)	0.514

Significant data are captured in bold

## Discussion

Fine regulation of cytokine levels is essential for a successful pregnancy. Improper balance in the levels of Th1 and Th2 cytokines has been known to contribute to recurrent miscarriage [6, 7]. Cytokine levels can be genetically controlled by its polymorphisms. As such, we postulate that polymorphisms in cytokine genes may influence the risk of recurrent miscarriage. To test this hypothesis, we investigated the association of eight selected cytokine gene polymorphisms with risk of recurrent miscarriage among Chinese women. Eight polymorphisms in five cytokine genes were selected, including *TNF* -238G > A, *TNF* -308G > A, *IL1B* -511 T > C, *IL1B* 3954C > T, *IL6* -174G > C, *IL6* -634C > G, *IL10* -1082A > G and *IFNG* 874A > T polymorphisms.

Among these polymorphisms, only *IL1B* -511 T > C polymorphism and *IL6* -634C > G polymorphism showed a statistically significant association with recurrent miscarriage risk. The *IL1B* -511 T > C polymorphism was found to be associated with an increased risk, with the mutant (CC) genotype having an OR of 1.377 (95% CI: 1.039–1.824). This finding was similar to a previous study in Korea [13], which showed that the polymorphism resulted in an increased risk by a magnitude of 1.826 (95% CI: 1.130–2.953). The authors from the Korean study also showed that the T to C substitution of the polymorphism contributed to a higher natural killer (NK) cell activity, causing an enhanced inflammatory state which is harmful for fetal development [13]. Apart from our work, there was another previous study which investigated the association of this polymorphism with recurrent miscarriage risk in the Chinese population [16]. The authors from this previous work indicated that the *IL1B* -511 T > C polymorphism was not associated with recurrent miscarriage risk, which was in contrast with our findings. However, it should be noted that great discrepancies exist in different subpopulations of China, and these discrepancies may affect the influence of the polymorphism on recurrent miscarriage risk. We studied the association between the polymorphism and recurrent miscarriage risk among residents of Zhejiang Province, while the previous work investigated the association in Jiangsu Province. This could explain the inconsistency in the finding obtained. Moreover, our study included a much larger number of participants compared to this previous work (1580 participants in the present work vs. 318 participants in the previous work), which provide a greater accuracy for our findings.

In this study, we also showed that the *IL6* -634C > G polymorphism was associated with a decreased risk of recurrent miscarriage, with the heterozygous (CG) genotype showed an OR of 0.614 (95% CI: 0.493–0.765) and the mutant (GG) genotype showed an OR of 0.414 (95% CI: 0.251–0.684). There were also two previous studies which investigated the association of this polymorphism with recurrent miscarriage risk, including one study from China [16] and one study from Japan [19]. Consistent with our study, both previous works showed that the polymorphism decreased the risk of recurrent miscarriage. The study from China found that the heterozygous and mutant genotypes showed OR values of 0.709 (95% CI: 0.438–1.148) and 0.894 (95% CI: 0.837–0.955) respectively, although only the latter was statistically significant, while another study from Japan showed that carriers of the G allele resulted in an OR of 0.46 (95% CI: 0.24–0.91).

We did not find any significant association of *TNF* -238G > A and -308G > A polymorphisms with risk of recurrent miscarriage. These two polymorphisms have been frequently investigated in the context of recurrent miscarriage, but the results obtained have been inconsistent [10–12, 14, 15, 18]. One of these previous studies was conducted in the Chinese population [15]. In this previous study by Liu et al. [15], the G allele of the *TNF* -238G > A polymorphism was found to be significantly more common in the cases. This means that the A allele was significantly associated with a decreased risk of recurrent miscarriage. Consistent with this previous work, our study showed that carriers of the A allele had a lower risk of recurrent miscarriage, although our observation did not reach statistical significance. Several other studies concurred with our findings [11, 12], while some others found that the A allele was significantly associated with increased risk of recurrent miscarriage [14, 18]. The reason behind these discrepancies is not well understood, although it is known that genetic polymorphisms affect disease risk differently in different populations. Besides, similar to our study, the work by Liu et al. [15] found no association between *TNF* -308G > A polymorphism with recurrent miscarriage risk in the Chinese population. This is consistent with a few previous reports [10, 11, 14, 18], but there was also one study which showed that the mutant AA genotype could increase the risk of recurrent miscarriage [12].

We also did not find an association of *IL1B* 3954C > T, *IL6* -174G > C, *IL10* -1082A > G and *IFNG* 874A > T polymorphisms with the risk of recurrent miscarriage. There was only one previous study which investigated the former (*IL1B* 3954C > T polymorphism) in recurrent miscarriage [16], and the result obtained was the same as our findings. On the other hand, there were two previous works which simultaneously reported the remaining three polymorphisms in recurrent miscarriage [10, 17]. One report from Brazil agreed entirely with our finding that there was no significant association observed between the polymorphisms and risk of recurrent miscarriage [10], while another work from India showed that the mutant alleles of the three polymorphisms increased the risk of recurrent miscarriage [17]. Besides these two previous reports, the *IL10* -1082A > G polymorphism had also been investigated in the South Korean population [14], and no statistically significant finding was observed. These discrepancies clearly showed that the polymorphisms influence the risk of recurrent miscarriage in a population-specific manner.

Our study had the strength in the statistical power. We included a total of 1580 participants (775 cases and 805 controls), which is the largest set of participants ever investigated for these eight polymorphisms. However, one limitation is that we did not adjust our findings by other potential confounding factors. This was due to the local legislation scenario, which disallowed us to access certain data of the patients. However, to minimize the influence of the confounding factors, we matched the participants carefully by age, and did not include participants with diabetes or hypertension, drug or alcohol abuse, and smoking habit. Despite this, future studies are needed to confirm our findings.

## Conclusions

We showed that the *IL1B* -511 T > C polymorphism was significantly associated with an increased risk of recurrent miscarriage, while the *IL6* -634C > G polymorphism was significantly associated with a decreased risk of recurrent miscarriage. These polymorphisms may potentially serve as important biomarkers for predicting recurrent miscarriage risk among Chinese. Future studies should embark on this research direction.

## Abbreviations

CI: Confidence interval
NK: Natural killer
OR: Odds ratio
PCR-RFLP: Polymerase chain reaction restriction fragment length polymorphism
Th: T helper
